# Optimal Product Placement

**DOI:** 10.1007/s11151-017-9575-y

**Published:** 2017-03-22

**Authors:** Chia-Ling Hsu, Rafael Matta, Sergey V. Popov, Takeharu Sogo

**Affiliations:** 1 School of Economics, Southwestern University of Finance and Economics, 555, Liutai Avenue, Wenjiang District, Chengdu, 611130 Sichuan People’s Republic of China; 2 University of Amsterdam, Plantage Muidergracht 12, Room M 4.23, 1018 TV Amsterdam, The Netherlands; 3 Cardiff Business School, Cardiff University, Aberconway Building, Colum Drive, Cardiff, CF10 3EU UK; 4 Osaka University of Economics, 2-2-8, Osumi, Higashiyodogawa-ku, Osaka, 533-8533 Japan

**Keywords:** Imperfect monopolistic competition, Internet trade intermediation, Menu choice, Search costs, Vertical differentiation, D21, D43, L11, L13

## Abstract

We model a market, such as an online software market, in which an intermediary connects sellers and buyers by displaying sellers’ products. With two vertically-differentiated products, an intermediary can place either: (1) one product, not necessarily the better one, on the first page, and the other hidden on the second page; or (2) both products on the first page. We show that it can be optimal for the intermediary to obfuscate a product—possibly the better one—since this weakens price competition and allows the sellers to extract a greater surplus from buyers; however, it is not socially optimal. The choice of which one to obfuscate depends on the distribution of search costs.

## Introduction

Many markets with intermediaries, such as online software markets, match buyers to sellers. It is often costly for buyers to locate the ones they most want, and this can affect the pricing policies of the sellers. Accordingly, intermediaries can increase sales by optimally choosing how to display their relevant products: e.g., to put one product on the front page and put another on the second page on the menu; or display one product close to the door and display another far from the door in the store.

We model the interactions between two sellers, a unit continuum of buyers, and a platform intermediary, in which any extensive search for desired products is costly.^1^ The sellers produce vertically differentiated products. The platform, which earns a fixed proportion of the profits from the sellers, selects which products to display on the front page; and the remaining products, if any, are displayed on the second page. We argue that the platform *strategically* decides to delegate products, possibly the better ones, to the second page to soften price competition, thereby extracting more buyer surplus.

In our main model with heterogeneous search costs across buyers, buyers can observe both the prices and qualities of both products, but cannot buy the second-page product before visiting the second page. We find that the shape of the distribution of search costs is a key determinant for the platform’s optimal arrangement of the products. For a large class of distributions of search costs, the platform hides the *better* product, which goes against the common findings in the literature.

We further extend our model to incorporate market segmentation: one form of horizontal differentiation. We find that the individual taste difference across products undermines the incentive to obfuscate the access to some products. Thus, when tastes are sufficiently heterogeneous, our previous prediction can be reversed: The platform shifts the focus from reducing price competition to targeting a greater audience.

### Online Platforms

As an illustration to our model, consider the market for apps for smartphones. According to Gartner (2013), the market for online mobile applications reached 64 billion downloads in 2012, generating over $18 billion in revenue. Table 1 indicates that the platforms—App Store, Google Play, BlackBerry World, and Windows Phone Store—receive an approximately fixed fraction of seller revenues.^2^ Thus, it would be reasonable to think that they are interested in maximizing the sum of the sellers’ revenues.

**Table 1:** Platforms

Name	Available apps	Seller’s share (%)	Fee
App Store	900,000 (July 2013)	56–71	$99/year
Google Play	1,000,000 (July 2013)	70	$25
BlackBerry World	120,000 (May 2013)	70	Free
Windows Phone Store	160,000 (May 2013)	70	$99/year

In the App Store, buyers usually make their purchase decisions from the products that are available on the front page of the platform. Hafner (2010) describes a typical consumer’s behavior: “A survey of iPhones, iPod Touch and Android users... found that people discover apps most often by browsing app stores. And even though the iTunes store is bloated with offerings, people tend to gravitate to the most popular.”

Platforms seem to have the technology (e.g., dynamic content loading) to display more products on the front page so that buyers can find products of interest to them without incurring additional search costs. The question is whether a platform *wants* to display more products on the front page and—if it chooses not to show more products on the front page—which products, of high or low quality, it wants to delegate to other pages.

### Related Literature

Broadly speaking, our study contributes to the vast literature on oligopoly theory that deals with ways to soften competition.^3^ Hotelling (1929) was the first to model this issue formally. d’Aspremont et al. (1979) use quadratic transportation costs in the Hotelling model, establishing that the firms choose maximum differentiation to soften price competition. Shaked and Sutton (1982) show that firms choose to differentiate their products vertically to soften price competition. Lancaster (1966) proposes the characteristics approach, which was later developed by Anderson et al. (1989).

The general lesson from these studies is that the relative importance of softening price competition and increasing market demand is the key determinant of product positioning: the degree of product differentiation (Belleflamme and Peitz 2015). Tirole (1988) identifies three forces that limit product differentiation: (i) the limited scope of price competition due to technology or regulation; (ii) the tendency to place products where the demand is; and (iii) the positive externalities between firms. Each of them increases the relative importance of increasing market demand over softening price competition.

In Sect. 3.1 we show that the platform may choose to display products separately using two pages to soften price competition. However, in Sect. 3.2, where we introduce a form of individual taste differences across products, we show that the platform would choose to display products together on a single page precisely because of (ii).

Important strands of the behavioral industrial organization literature study how the presence of behavioral consumers—who search too little, stick too much to past choices, and/or have biased expectations about their own future choices—can lead to positive markups even in a competitive environment (see reviews by Grubb 2015a, b). Although we too find that positive markups are offered in equilibrium with competition between sellers, we assume that all agents are *homo economicus*.

Our study more directly contributes to the intentional obfuscation literature. Ellison and Wolitzky (2012) develop the idea of Stahl (1989) to consider a form of obfuscation: Sellers may *choose* to increase the search costs to deter buyers from searching, so that the sellers can charge prices that are close to monopolistic ones (see also Wilson 2010).

Another interpretation of obfuscation comes from Ellison (2005): He studies an add-on price model, where sellers produce vertically-differentiated products, while advertising only low-price products to attract buyers, who will be induced to purchase the upgraded version, the high-quality product. This view is empirically supported by Ellison and Ellison (2009).

Hagiu and Jullien (2011) suggest that the platform might strategically increase the effective search costs by intentionally reducing the efficiency of searching. However, in their model the platform intentionally mismatches consumers with their less preferred goods, whereas our platform behaves identically regardless of the type of buyer (see also White 2013).

Empirical studies about search obfuscation acknowledge the significance of search costs, even when these costs involve merely moving one’s eyes one line down a list.^4^ Koulayev (2014) finds empirical evidence for the dependence of price elasticity on the size of search costs in online hotel bookings. McDonald and Wren (2013) find empirical evidence that the practice by online insurance sellers of posting multiple prices under different brand names is consistent with search obfuscation. In contrast, we theoretically show that the platform, not the sellers, obfuscates a product to soften price competition.

In the directed search literature, Weitzman (1979) asks how an agent would choose the order of sampling for a set of products [see also Wolinsky (1986) and Zhou (2011) for differentiated products]. Arbatskaya (2007) studies the pricing rules of homogeneous companies when a sampling sequence is given exogenously. Our paper differs in two ways: first, our products are heterogeneous; and second, the ordering is chosen by the platform, not the buyers.

In a more closely related paper, Armstrong et al. (2009) analyze prominence in the sense that a prominent firm is one sampled first by consumers. They find the highest-quality firm earns the greatest profit by becoming prominent, implying that consumers sample products in order of quality. In contrast, we find that a platform may display the worse product first.

Another closely related paper is by Song (2016), who considers the manipulation of product positions on different pages in a similar framework. Unlike our paper, his focuses on horizontal differentiation and shows that goods with more ambiguous characteristics—goods that provide more uncertain utility—are better kept on the front page.

In Baye and Morgan (2001), advertisers pay fees to the platform to advertise their prices and consumers pay for access to these prices. The platform sets advertising fees sufficiently high to avoid excessive participation and thus excessive price competition between advertisers, which would reduce the rents that the platform can extract from advertisers. In Kamenica (2008), the platform chooses the sequence of products to show to consumers in order to affect consumers’ beliefs about the availability of products. However, we assume that consumers are knowledgeable about their available options.

Athey and Ellison (2011) study position auctions in which advertisers bid for sponsored-link positions—with values to advertisers that are contingent on the sales to consumers—and consumers rationally infer the qualities of links from the ordering of those links. Athey and Ellison focus on equilibria in which bids are increasing in quality: Sponsored-links are ordered from highest quality to lowest. However, we show that a platform may display the worse product first.

Our paper is also related to the Mussa and Rosen (1978) model of quality choice by a monopolist. Donnenfeld and White (1990) establish that increasing the quality difference by reducing the quality of the lower-quality product will increase the monopolist’s profit, since it will make it more difficult for buyers with high willingness-to-pay for quality to switch to the lower-quality product, inducing price discrimination.

Similarly, the “damaged goods” literature (Deneckere and McAfee 1996) establishes that with heterogeneity in consumers’ private valuations of products, selling the low-quality version in addition to the high-quality version enables the seller better to segment consumers and induce price discrimination. In our model, by contrast, having the low-quality product present can benefit the platform when two pages are used, but this is because it reduces price competition.

The rest of the paper is organized as follows: Sect. 2 presents a model of product placement with homogeneous search costs. Section 3 deals with heterogeneous search costs across buyers. Section 3.2 incorporates market segmentation. Section 4 concludes. Appendix contains proofs that are omitted from the text.

## The Menu Choice Problem

In this section, we use a simple one-period model to examine how choices by a platform interact with the prices that are set by two sellers, who face a continuum of buyers of measure one. All agents are risk neutral.

We assume that the platform profit is a *fixed* share of the sum of the sellers’ profits.^5^ Thus, we can normalize the platform profit as the sum of the sellers’ profits. Consequently, in what follows, we envisage the platform’s arranging the products to maximize the total profits of the sellers.

The assumption that the platform profit is a fixed share of the sellers’ profits reflects the practices of online platforms summarized in Table 1. Moreover, as discussed in Footnote 2, not only online software markets but also other online markets such as Amazon and eBay fit well with this assumption as long as its analysis is within the same category (e.g., within the book department on Amazon). This assumption makes it harder to extend our model to department stores and shopping malls:^6^ The operators of such stores may charge different percentages of sales to different store tenants.^7^

Each seller $i\in \{1,2\}$ produces product *i* of quality $r_{i}>0$ at zero marginal cost^8^ and sets its price *after* their product positions are assigned by the platform. We denote $\varDelta =r_{1}-r_{2}$. Without loss of generality, product 1 is of higher quality: $\varDelta >0$. We assume that qualities are exogenously given, excluding the possibility that sellers adjust their qualities after product positions are arranged. This reflects the fact that qualities are often more difficult to adjust than are prices in response to an arrangement of products.

Buyers obtain utility $r_{i}-p_{i}$ from buying and consuming product *i* of quality $r_{i}$ upon paying price $p_{i}$. Buyers must pay a search cost $c>0$ if they visit the second page. They can also quit and collect the reservation utility of 0. Thus, a unique socially efficient outcome is that all buyers purchase product 1 on the front page.

After observing the product qualities $r=\left( r_{1},r_{2}\right)$, the platform decides on product placement. It can place products on one or on two pages. The choice of the platform is a probability distribution over the arrangement of products. We denote this choice by $\varTheta =\{\theta _{1},\theta _{2},\theta _{12}\}\in \varDelta ^{3}$: a 3-dimensional simplex, where $\theta _{s}$ is the probability that a random buyer will encounter the set of products *s* on the front page.

That is, $\theta _{12}$ is the probability of having both products on the front page (producing effectively no second page); and $\theta _{i}$ is the probability of having only product *i* on the front page, with the other product available only on the second page. Thus, the set of products on the front page that each buyer faces is independently determined on the basis of $\varTheta$.

When buyers visit the front page, they observe the price(s) and quality(-ies) of the product(s) on the front page, and they also know the arrangement of products on the second page. For example, if $\theta _{1}=1$, then the buyers know that the product on the front page has quality $r_{1}$ and that the product on the second page has quality $r_{2}$. Buyers can purchase only the products on the pages that they have visited.^9^ We impose the following tie-breaking rule: When buyers are indifferent, they purchase a product on the page where they end up; and if there are two products on the same page, they purchase the product of better quality: product 1.

The timing of the game is as follows: The platform observes the qualities and decides on product placements $\varTheta$. As a response to the platform’s choice, the sellers set the prices.^10^ Buyers enter the first page, deciding whether to buy and whether to visit the second page.

The equilibrium is the collection of $\left( \varTheta ^{*},p^{*},q^{*},\mu ^{*}\right)$ that satisfies the following:

- $\varTheta ^{*}(r)$ maximizes the sum of the sellers’ profits, given $\left( p^{*},q^{*},\mu ^{*}\right)$;
- $p^{*}(r,\varTheta )=\left( p_{1}^{*},p_{2}^{*}\right) \in R^{2}$ is a set of prices that the sellers charge given $(r,\varTheta ),$ such that price $p_{i}^{*}$ is the seller *i*’s best response to $\varTheta$ and $p_{-i}^{*}$;
- $q^{*}(\mu ,c)\in \{0,1\}$ is the browsing decision of buyers: $q^{*}(\mu ,c)=1$ if a buyer visits the second page, and $q^{*}(\mu ,c)=0$ otherwise, when the buyer incurs search cost *c* and holds a belief $\mu$ about the surplus that is provided by the second-page product, such that $q^{*}(\mu ,c)$ maximizes the buyer’s utility; and
- beliefs $\mu ^{*}(\varTheta )$ are consistent with all actions by all agents on the equilibrium path.

### Homogeneous Search Costs

In this subsection, we assume that all buyers have the same search cost $c>0$. In order to understand the motivation of Assumption 1 below, let us first assume that buyers do not know the price of the second-page product unless they have visited the second page.

Consider the first-best scenario: $\theta _{12}=1$. The standard Bertrand competition argument yields $p_{1}=\varDelta$ and $p_{2}=0$; and the platform revenue is $\varDelta$. As the following proposition states, the platform chooses $\theta _{1}=1$ in which both sellers charge the monopoly price. Thus, the *Diamond paradox* (Diamond 1971) arises if buyers do not know the price on the second page.

To see this, suppose $\theta _{1}=1$. Note that there is no equilibrium in which a positive fraction of the buyers visits the second page—because seller 1 can always undercut its price to capture the entire share. Thus, in any robust equilibrium,^11^ seller 2 charges its monopoly price to the buyers who visit the second page by mistake; that is, $p_{2}=r_{2}$. Since no buyer visits the second page, seller 1 charges the monopoly price $r_{1}.$ Moreover, it is optimal for buyers not to visit the second page, given $p_{2} =r_{2}$. We are now ready to state the first part of the proposition:

#### **Proposition 1**

*When* $\theta _{1}=1$, *the only robust equilibrium is that* $p_{1} = r_{1}$, $p_{2} = r_{2}$, *and buyers do not visit the second page; the platform receives a revenue of* $r_{1}$. *Moreover,* $\theta _{1}=1$ *is the unique equilibrium: All other choices of* $\varTheta$ *but* $\theta _{1}=1$ *yield less than* $r_{1}$ *to the platform*.

Proposition 1 demonstrates that the Diamond paradox arises: because the buyers expect both sellers to set monopoly prices—if anyone should visit the second page, the second-page seller cannot credibly commit to rewarding the switchers, and would instead succumb to the temptation to maintain the monopoly price so that *after* searching the buyers become indifferent between buying product 2 and not—they have no incentive to do a costly search.

Thus, even if we deal with heterogeneous search costs—because the robust equilibrium does not depend on *c*—the equilibrium outcome is that all buyers decide not to go to the second page. To avoid this version of the Diamond paradox, we impose the following assumption:

#### **Assumption 1**

Buyers know the price and the quality of the product on the second page.

Buyers know both the prices and qualities of both products, but cannot buy the second-page product before visiting the second page. In this sense, the costly search in this model is not to *learn* about products, but rather to *locate* products.

Our analysis extends to a case in which buyers do not know the price on the second page so long as: (1) the second-page seller publicly promises its price in advance; (2) an infinitesimal fraction of buyers visit the second page at the beginning of the trading day; and (3) should the seller in question break its promise, these buyers publicly disclose it, which hurts the seller substantially enough to make it refrain from doing so.^12^ Assumption 1 yields Proposition 2:

#### **Proposition 2**

*Under Assumption* 1, *the equilibrium is that* $\theta _{1}=1$, *the buyers do not go to the second page, and* $\left( p_{1},p_{2}\right) =\left( r_{1},p_{2}^{*}\right)$, $\forall p_{2}^{*}\ge 0$ *if* $c\ge r_{2}$; $\left( p_{1},p_{2}\right) =\left( \varDelta +c,0\right)$ *if* $c<r_{2}$.

This illustrates that the platform can soften price competition and enjoy a greater profit than that under Bertrand competition, $\varDelta$, by displaying only the better product on the front page. Moreover, as the search cost becomes smaller, it becomes more difficult for the platform to soften price competition and extract the buyers’ surplus.

## Heterogeneities

In this section, we analyze the effects of introducing heterogeneous search costs on the optimal placement decision by the platform and its associated welfare outcomes. Search costs are distributed i.i.d. according to a distribution $G(\cdot )$ and a continuous density $g(\cdot )$ with full support on $\left[ 0,\bar{c}\right]$. To ensure the uniqueness of the equilibrium (which we will check later), we assume that $G(\cdot )$ and $g(\cdot )$ satisfy the following assumption:^13^

### **Assumption 2**

$\frac{G(c)}{g(c)}$ is increasing, and $\frac{1-G(c)}{g(c)}$ is decreasing.

Assumption 2 is satisfied when $g(\cdot )$ is log-concave [see Theorems 1 and 3 of Bagnoli and Bergstrom (2005) for proof].

Further, in order to simplify our future analysis—because the quitting option affects the equilibrium behaviors in a not very interesting way, as in Proposition 2—we assume that the restrictions imposed by the quitting option do not bind in equilibrium. Since the prices turn out to be independent of $r_{2}$, the utility from a purchase can be written as:

$$\begin{aligned} \max \{\underbrace{r_{2}+\varDelta }_{r_{1}}-p_{1},r_{2}-p_{2}\}. \end{aligned}$$

Thus, assuming a sufficiently large $r_{2}$ will render the quitting option irrelevant. We will refer to this as *the quitting-option irrelevance* in the analysis below.

### Heterogeneous Search Costs

With heterogeneous search costs, showing both products on the front page is still the first-best and the subsequent optimal behavior is the same as before: It is socially inefficient for a positive mass of buyers to pay search costs and purchase a product on the second page. However, the platform can now soften price competition by concealing one product. Would the platform prefer to conceal one product, possibly the better one, on the second page?

First consider $\theta _{1}=1$: The platform puts only product 1 on the front page. Then, buyers with low search costs purchase from the second page, and collect $r_{2}-p_{2}-c$ instead of buying product 1 and collecting $r_{1}-p_{1}$. Thus, there exists a cutoff search cost, $c_{1}^{*}$, such that buyers with search cost $c_{1}^{*}$ are indifferent between visiting the second page and not:

$$\begin{aligned} r_{1}-p_{1}=r_{2}-p_{2}-c_{1}^{*}\Rightarrow c_{1}^{*}=p_{1}-p_{2}-\varDelta . \end{aligned}$$

Observe that more buyers go to the second page as $$p_{1}$$ increases or $$p_{2} $$ decreases. The profit of seller 1 given $$p_{1}$$ is:

$$\begin{aligned} \pi _{1}(p_{1})=p_{1}(1-G(c_{1}^{*}))=p_{1}(1-G(p_{1}-p_{2}-\varDelta )); \end{aligned}$$

and the seller 2’s profit given $p_{2}$ is

$$\begin{aligned} \pi _{2}(p_{2})=p_{2}G(c_{1}^{*})=p_{2}G(p_{1}-p_{2}-\varDelta ). \end{aligned}$$

The first-order condition of seller 1 implies that in response to a marginal increase in the price of product 1, the revenue increase from the remaining customers, whose search costs are greater than $c_1$, is exactly compensated by the revenue loss, from losing marginal customers of amount $g(c_1^*)$: $1-G(c_{1}^{*})=g(c_{1}^{*})p_{1}^{*}$. Therefore,

$$\begin{aligned} p_{1}^{*}=\frac{ 1-G(c_{1}^{*})}{g(c_{1}^{*})}. \end{aligned}$$

The corresponding first-order condition for seller 2 yields $$p_{2}^{*}= G(c_{1}^{*})/g(c_{1}^{*})$$. Therefore, the cutoff search cost $$ c_{1}^{*}$$ induced by the equilibrium prices $$\left( p_{1}^{*},p_{2}^{*}\right) $$ solves

1 $$\begin{aligned} c_{1}^{*}=p_1(c_1^*)-p_2(c_1^*)-\varDelta =\underbrace{\frac{1-2G(c_1^*)}{g(c_1^*)}}_{\equiv Q(c_{1}^{*})}-\varDelta . \end{aligned}$$

Assumption 2 ensures that the right-hand side of (1) is continuous and decreasing in $c_{1}^{*}$. Thus, the solution to (1) is unique whenever it exists. The solution exists if $1/g(0)>\varDelta$; i.e., if $\varDelta$ or the density at $c=0$ is sufficiently low.

Similarly, consider $\theta _{2}=1$ and let $c_{2}^{*}$ denote the associated cutoff search cost. $c_{2}^{*}$ solves:

$$\begin{aligned} r_{1}-p_{1}-c_{2}^{*}=r_{2}-p_{2}\Rightarrow c_{2}^{*}=p_{2}-p_{1}+\varDelta . \end{aligned}$$

As in the previous case, one can obtain $p_{1}^{*}=G(c_{2}^{*})/g(c_{2}^{*})$, $p_{2}^{*}=(1-G(c_{2}^{*}))/g(c_{2}^{*})$, and

2 $$\begin{aligned} c_{2}^{*}=p_2(c_2^{*})-p_1(c_2^{*})+\varDelta =Q(c_{2}^{*})+\varDelta . \end{aligned}$$

The fixed point exists if $1/g(\bar{c})>\varDelta -\bar{c}$.

*Quantities* $c_{1}^{*}<c_{2}^{*}$: *More* people visit the second page if the better good is there. To see this, suppose otherwise. Then, by (1) and (2), $Q(c_{1}^{*})>Q(c_{2}^{*})$ must hold, which contradicts $c_{1}^{*}\ge c_{2}^{*}$ and $Q(\cdot )$ being a decreasing function by Assumption 2. Therefore, $c_{1}^{*}<c_{2}^{*}$.

To see the logic behind $c_{1}^{*}<c_{2}^{*}$: First suppose that the sellers on the same page set the same price regardless of whether the better or the worse product is on the front page. Then, a buyer with search cost $c_{1}^{*}$ is indifferent between visiting the second page and not, when the worse product is on the second page: $\theta _{1}=1$. This marginal buyer would strictly prefer to visit the second page if the better product is on the second page. The second-page seller under $\theta _{2}=1$ has the quality advantage over the second-page seller under $\theta _{1}=1$, which allows it to match its price to that of the second-page seller under $\theta _{1}=1$. Therefore, $c_{1}^{*}<c_{2}^{*}$ must follow.

*Prices* Prices reflect product qualities. The price of product 1 is increasing in $\varDelta$, and the price of product 2 is decreasing in $\varDelta$, regardless of their placements: because $c_{1}^{*}(\varDelta )$ is decreasing, $c_{2}^{*}(\varDelta )$ is increasing, and the hazard rates that set the prices are monotone.

Let $p_{1}$ and $p_{2}$ be the prices of product 1 and 2 under $\theta _{1}=1$, and let $p_{1}^{\prime }$ and $p_{2}^{\prime }$ be the prices of product 1 and 2 under $\theta _{2}=1$. By Assumption 2 and $c_{1}^{*}<c_{2}^{*}$, it follows that the better product is priced higher if located on the same page: $p_{1}=(1-G(c_{1}^{*}))/ g(c_{1}^{*})$ is *higher* than $p_{2}^{\prime }=(1-G(c_{2}^{*}))/g(c_{2}^{*})$; and $p_{1}^{\prime }=G(c_{2}^{*})/g(c_{2}^{*})$ is *higher* than $p_{2}=G(c_{1}^{*})/g(c_{1}^{*})$.

When product 1 is on the front page, product 1 has higher quality than product 2 and does not require any search cost. Thus, most customers find product 1 more attractive: $c_{1}^{*}$ is smaller than the median; that is, $G(c_{1}^{*})<1-G(c_{1}^{*})$. Therefore, $p_{2}<p_{1}$.

However, when product 2 is on the front page, product 2 does not require any search cost, but has lower quality than product 1. Thus, $p_{1}^{\prime }$ can be higher than $p_{2}^{\prime }$.

Nonetheless, if $\varDelta$ is sufficiently small, most customers find product 2 more attractive—$c_{2}^{*}$ becomes less than the median—because it does not require search and the quality disadvantage is small, yielding $p_{1}^{\prime }<p_{2}^{\prime }$. Thus, we can summarize the above discussion as follows:

#### **Claim 1**

*When* $\varDelta$ *is sufficiently small*, $p_{2}<p_{1}^{\prime }<p_{2}^{\prime }<p_{1}$ *holds*.

*Platform Profits* Under $\theta _{1}=1$, $p_{1}^{*}=(1-G(c_{1}^{*}))/ g(c_{1}^{*}),$ and the sales volume of product 1 is equal to the total of buyers who do not go to the second page, or $(1-G(c_{1}^{*}))$; $p_{2}^{*}=G(c_{1}^{*})/g(c_{1}^{*}),$ and the sales volume of product 2 is equal to $G(c_{1}^{*})$. Therefore, the platform profit under $\theta _{1}=1$ is $\pi (c_{1}^{*})=( (1-G(c_{1}^{*}))^{2}+(G(c_{1}^{*}))^{2})/g(c_{1}^{*})$. Similarly, the platform profit is $\pi (c_{2}^{*})=( (1-G(c_{2}^{*}))^{2}+(G(c_{2}^{*}))^{2})/g(c_{2}^{*})$ under $\theta _{2}=1$.

Note that under $\theta _{12}=1$, the platform charges $\varDelta$ for product 1 and 0 for product 2, inducing *everyone* to buy the better product, and thereby earning a profit of $\varDelta$. In contrast, under $\theta _{1}=1$, displaying products on separate pages softens price competition and increases the prices of both products. However, those who visit the second page purchase the cheaper product; that is, *not everyone* buys the better product, which is more expensive.

When $\varDelta$ is sufficiently large, the cost of $\theta _{1}=1$—that not everyone buys the expensive better product—outweighs the benefit of softening price competition. Thus, it is optimal for the platform to display both products on the front page and induce everyone to buy the expensive better product, so that the platform secures a profit of $\varDelta$.

As the quality difference $\varDelta$ becomes smaller, the difference between the prices of the better and the worse products becomes smaller; the cost of $\theta _{1}=1$, that not everyone buys the expensive better product, becomes smaller. Thus, when $\varDelta$ is small, the benefit of $\theta _{1}=1$ to soften price competition outweighs the cost: Showing only one product yields a higher profit than showing both products on the front page.

In the extreme case of $\varDelta =0$, the platform earns zero profits by showing both products on the front page, while it earns positive profits from both products by showing them on separate pages. By the continuity of profits in $\varDelta$, it follows that when $\varDelta$ is sufficiently small, showing only one product yields a higher profit than showing both on the front page.

We first state the sufficient condition for the optimality of $\theta _{1}=1$ under the assumption that $\varDelta$ is sufficiently small so that, together with the quitting-option irrelevance, it ensures that either $\theta _{1}=1$ or $\theta _{2}=1$ yields the highest platform profit:

#### **Proposition 3**

*For sufficiently small* $\varDelta$,^14^ *if* $g(\cdot )$ *is symmetric, the platform is better off showing only product* 1 *on the front page*.

#### *Example 1*

15 Suppose that *g* is uniform on $\left[ 0,1\right]$ to illustrate Proposition 3. For $\varDelta <1$, which is required for solutions $c_{1}^{*}$ and $c_{2}^{*}$ to exist, $c_{1}^{*}=1/3-\varDelta /3$; $c_{2}^{*}=1/3+\varDelta /3$; $\pi \left( c_{1}^{*}\right) =\left( 2\varDelta ^{2} +2\varDelta +5\right) /9;$ and $\pi \left( c_{2}^{*}\right) =\left( 2\varDelta ^{2}-2\varDelta +5\right) /9$. Thus, $\pi \left( c_{1}^{*}\right) >\pi \left( c_{2}^{*}\right)$ and $\pi \left( c_{1}^{*}\right) >\varDelta$ hold for $\varDelta <1$; that is, $\theta _{1}=1$ is optimal. As explained above, the incentive to soften price competition increases as $\varDelta$ becomes smaller. Thus, for $\varDelta <1$ in this example, the platform chooses to display only one product on the front page.

The symmetry, however, is not necessary for the optimality of $\theta _{1}=1$.

#### *Example 2*

Suppose the distribution of *c* is *beta*(1.05, 10), which is positively skewed:

$$\begin{aligned} g(c)=\frac{\varGamma \left( 11.05\right) }{\varGamma \left( 1.05\right) \varGamma \left( 10\right) }c^{0.05}(1-c)^{9}, \quad c \in (0,1). \end{aligned}$$

Following the above analysis yields that it is optimal for the platform to display only product 1 for $\varDelta$ above approximately 0.09.^16^ However, it is optimal to delegate product 1, the better one, to the second page for $\varDelta < 0.09$.

Let us elaborate why the platform may want to delegate the better product to the second page and that the result is not restricted to the particular density function that is considered in Example 2—the key characteristic required for the result is positive skewness.

#### **Claim 2**

*For the platform to prefer to show only product* 2 *on the front page, one would need a positively skewed distribution of search costs*.

Suppose $\varDelta$ is sufficiently small, so that $p_{2}<p_{1}^{\prime }<p_{2}^{\prime }<p_{1}$ holds: The good on the second page is cheaper than the good on the front page. Buyers with $c<c_1^*$ are *searchers*: Their search cost is so low that they search regardless of product placements. Buyers with $c \in [c_1^*, c_2^*]$ are *switchers*: Their search cost is intermediate so that they do not search when product 1 is displayed first, but switch to search when product 2 is displayed first: They always buy product 1. Buyers with $c>c_2^*$ are *stayers*: Their search cost is so high that they stay and buy whichever product is displayed first regardless of product placements.

When product 1 is on the front page, searchers buy product 2, and switchers and stayers buy product 1; when product 2 is on the front page, searchers and switchers buy product 1, and stayers buy product 2. This means that putting product 2, instead of product 1, on the front page affects the platform’s profits in three ways:

- The profits from searchers increase by $(p_1'-p_2)G(c_1^*)$ because they buy the expensive better product from the second page;
- The profits from stayers decrease by $(p_1-p_2')(1-G(c_2^*))$ because they buy the cheaper worse product from the front page; and
- The profits from switchers decrease by $(p_1-p_1')(G(c_2^*)-G(c_1^*))$ because the better product becomes cheaper and they always buy the better product regardless of product placements.

Thus, the platform earns more from searchers and less from stayers and switchers. Showing the worse product first yields higher profit if there are many searchers; i.e., if density $g(\cdot )$ is positively skewed: Example 2 is based on such density.

To summarize, when $\varDelta$ is small, the cost of obfuscation—that not everyone buys the expensive better product—becomes smaller; therefore, because of the benefit of softening price competition, using two pages is optimal. Thus, the relevant choice for the platform is reduced to $\theta _{1}=1$ or $\theta _{2}=1$ for sufficiently small $\varDelta$.

If the distribution of search costs is positively skewed, regardless of which product is displayed on the second page, many buyers go to the second page. That is, the cost of $\theta _{1}=1$—that fewer buyers purchase the expensive better product—is high. Thus, it can be optimal to delegate the better product to the second page and induce many buyers to purchase the expensive better product.

### Market Segmentation

This subsection illustrates that the interaction of *horizontal* heterogeneity and vertical heterogeneity can *reverse* the results that were obtained under vertical heterogeneity only. To allow for some degree of horizontal differentiation, in the spirit of Wolinsky (1986) we assume that the population is separated into groups so that some buyers cannot consume or they derive zero utility from one of the products.^17^ The buyers are separated into three groups, indexed by *i*, with mass of $\mu _{i}$ such that $\mu _{1}+\mu _{2}+\mu _{3}=1$, as summarized in Table 2.

**Table 2:** Preference structure

Group #	Can consume	
*i*	Good 1	Good 2
1	Yes	No
2	No	Yes
3	Yes	Yes

Thus, when the prices of both goods are the same, group 3 buyers find product 1 more attractive, but group 2 buyers purchase product 2.

Under $\theta _{1}=1$, the buyers of group 1 buy product 1 on the front page. Group 3 decides whether to visit the second page on the basis of search costs; the indifferent buyer is given by $c_{1}^{*}=p_{1}-p_{2}-\varDelta$. Thus, seller 1’s problem is:

$$\begin{aligned} \max _{p_{1}}p_{1}\left( \mu _{3}(1-G(p_{1}-p_{2}-\varDelta ))+\mu _{1}\right) . \end{aligned}$$

The first-order condition yields $p_{1}^{*}=(\mu _{3}(1-G(c_{1}^{*}))+\mu _{1})/(\mu _{3}g(c_{1}^{*}))$. Analogously, $p_{2}^{*}=(\mu _{3}G(c_{1}^{*})+\mu _{2})/(\mu _{3}g(c_{1}^{*}))$. The equilibrium cutoff search cost is, therefore, a solution to the equation:

$$\begin{aligned} c_{1}^{*}=Q\left( c_{1}^{*}\right) -\varDelta +\frac{\mu _{1}-\mu _{2}}{ \mu _{3}g(c_{1}^{*})}. \end{aligned}$$

Similarly, under $\theta _{2}=1$, the equilibrium cutoff search cost solves

$$\begin{aligned} c_{2}^{*}=Q\left( c_{2}^{*}\right) +\varDelta -\frac{\mu _{1}-\mu _{2}}{\mu _{3}g(c_{2}^{*})}. \end{aligned}$$

Notice that, compared to the corresponding conditions (1) and (2) for the case without horizontal differentiation, we have the additional terms $(\mu _{1}-\mu _{2})/(\mu _{3}g(c_{1}^{*}))$ and $-(\mu _{1}-\mu _{2})/(\mu _{3}g(c_{2}^{*}))$ for $c_{1}^{*}$ and $c_{2}^{*}$, respectively. The smaller is the proportion $\mu _{3}$ of buyers who switch between product 1 and 2, the stronger is the effect of the difference in $\mu _{1}-\mu _{2}$ (the relative sizes of the groups of buyers who purchase products 1 and 2, respectively) on the size of the shift away from the case without horizontal heterogeneity.^18^

Consider the case in which the density $g(\cdot )$ is such that it is optimal for the platform to choose $\theta _{1}=1$ when $\mu _{1}=\mu _{2}$. If $\mu _{1}>\mu _{2}$, the results in Sect. 3.1 are reinforced: The relative attractiveness of $\theta _{1}=1$ is magnified.

However, if $\mu _{1}<\mu _{2}$, $\theta _{2}=1$ can become optimal. Even though the distribution of the search costs calls for placing product 1 on the front page, the lack of demand for product 1 may induce too many buyers to visit the second page. Then, the platform can improve its revenues by placing the worse product on the front page to earn higher markups from its greater audience. When $\mu _{2}$ is large, the platform’s focus shifts to increase $p_{2}$ as much as possible, making $\theta _{2}=1$ optimal.

Similarly, when the density $g(\cdot )$ is such that it is optimal for the platform to choose $\theta _{2}=1$, the results in Sect. 3.1 can be *reversed* if $\mu _{1}>\mu _{2}$.

Returning to Example 2, we can see that the size of $\frac{\mu _{1}-\mu _{2}}{\mu _{3}}$ is key in the reversal. This is the reason why we ventured into a discussion of horizontal differentiation. A monopolist can obfuscate a product and extract more surplus from buyers by using vertical differentiation.

Less vertical differentiation $\varDelta$ increases the incentive of the platform to soften competition, which results in an inefficient outcome. But horizontal differentiation, even in the crude form analyzed here, undermines the incentive to soften competition, which may result in reversing the prediction of Sect. 3.1. Greater market heterogeneity—$1-\mu _{3}$—can motivate the platform to display *both* products on the front page.

## Conclusion

We study a platform’s choice of which vertically-differentiated products to display on the front page, when it is costly for consumers to make an extensive search and the platform receives a fixed fraction of the revenues earned by the sellers.

We first show that when buyers’ search costs are homogeneous, they do not visit the second page, and they will purchase the better of two products. We then extend this analysis to heterogeneous search costs and find that, when quitting is not a relevant option, the platform has significant incentives to hide one product on the second page, but not necessarily the worse one.

The optimality of putting either the higher- or lower-quality product on the front page can explain the difference between the platforms’ methods of ranking apps. If the platform ranks by customers’ satisfaction, it displays the better product on the front page. If the platform ranks by the number of downloads, however, a worse product can be displayed first. Thus, the latter method could be optimal if the distribution of search costs is positively skewed.

We further extend our analysis to market segmentation. We show that—especially when market heterogeneity is great—the form of horizontal differentiation can reverse the prediction of our model because it shifts the platform’s focus from reducing price competition to targeting a greater audience. We can interpret that, in actual online stores, the front pages contain many products precisely because these products are horizontally-differentiated and unlikely to compete with each other.

Moreover, we expect our main model to fit better with markets that face less heterogeneity in taste (e.g., the market for navigation software, and the market for media players). For a consumer who looks for a program in such a market, all the products on the front page that do not belong to the necessary category can be considered as quitting options. Looking for the model’s “product on the second page” for such a consumer requires not only clicking on the link, but also formulating the search query, and filtering the results there.

When the platform hosts more than two products, it can benefit from adding more pages. It benefits not only from increasing the effective search costs by using only the first and the last page, but also from segmenting the buyer population into smaller submarkets, thereby extracting more surplus from buyers. Welfare is likely to suffer because of higher search costs and allocation inefficiency, but the platform can only benefit from adding more pages.

Our model may be tested in a price-volatile market such as the Apple App Store. Observing individuals’ buying behavior given the options available on the front and second pages would give us information about the distribution of search costs. Moreover, by observing changes in positioning across pages as responses to updates, we can test whether the platform analyzes product qualities when deciding its product placement.

## Figures and Tables

**Fig. 1:**
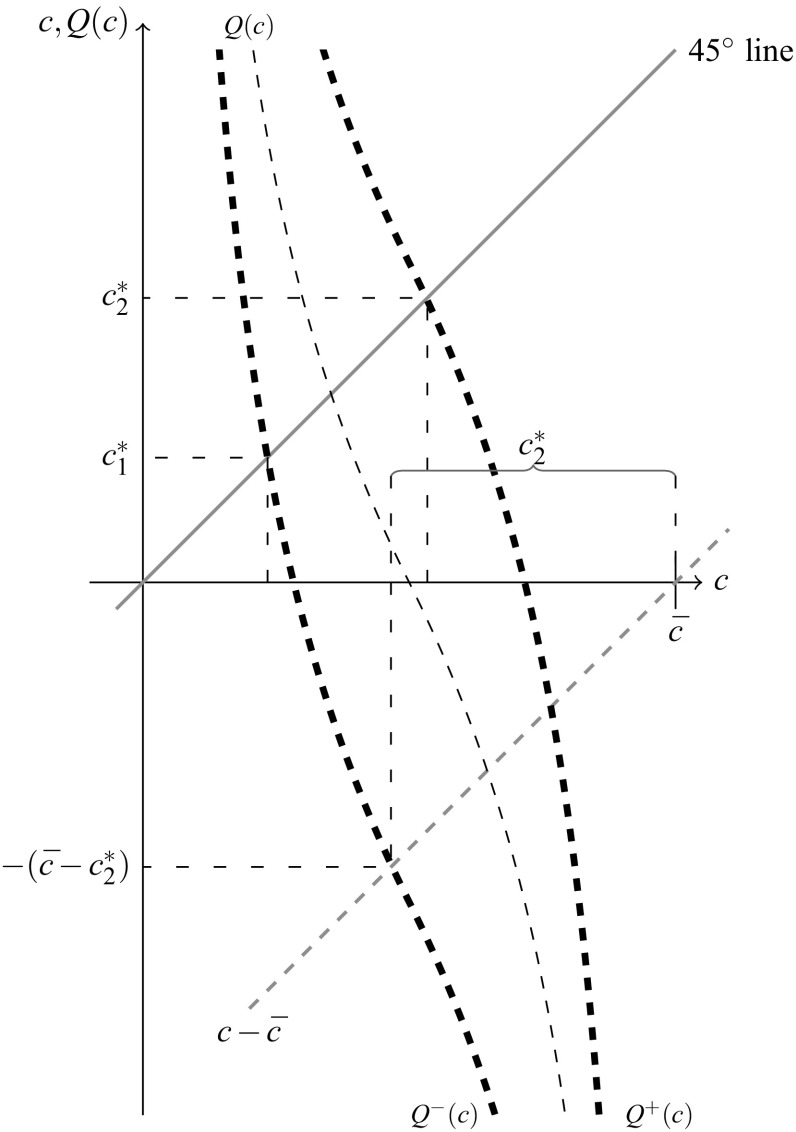
Under symmetry, obfuscating good 2 is better than obfuscating good 1

## Appendix: Proofs

### *Proof*

*(Proposition* 1*)* To establish the latter part of the proposition, we first show that the platform revenue cannot exceed $r_{1}$ in any equilibrium, and then show that the other menu choices besides $\theta _{1}=1$ deliver strictly less than $r_{1}$.

${r_{1}}$ *is the maximum revenue* To have a total revenue higher than $r_{1}$, at least one of the sellers must charge a price higher than $r_{1}$. However, no buyer will purchase from such a seller. Therefore, it is not possible for the platform to receive a revenue higher than $r_{1}$.

${\theta _{1}=1}$ *is the best* There are three pure strategy menu choices. We have shown that $\theta _{1}=1$ yields a revenue of $r_{1}$ in the unique robust equilibrium. When $\theta _{12}=1$, it immediately follows that $p_{1}=\varDelta$ and $p_{2}\ge 0$. All buyers purchase from seller 1, yielding a revenue of $\varDelta$. When $\theta _{2}=1$, it follows that a unique robust equilibrium is $p_{2}=r_{2}$ and $p_{1}=r_{1}$, and buyers believe that it is not worthwhile to go to the second page, yielding a revenue of $r_{2}<r_{1}$. Therefore, $\theta _{1}=1$ is the only menu choice that generates $r_{1}$ in robust equilibria (including mixed strategies). $\square$

### *Proof*

*(Proposition* 2*)* We first show that given $\theta _{1}=1$, the stated strategies are best responses to each other. When $r_{2}\le c$, seller 2 cannot attract any buyer even by setting $p_{2}=0$ regardless of $p_{1}$. Therefore, seller 1 will act as a monopolist.

Suppose $$r_{2}>c$$. Seller 1’s best response to $$p_{2}=0$$ is to set the maximum $$p_{1} $$ such that $$p_{1}\le \varDelta +c$$ and $$p_{1}\le r_{1}$$; that is, to set $$p_{1}=\varDelta +c$$. Seller 2’s best response to $$p_{1}=\varDelta +c$$ is to attract no buyer, because it must earn a negative profit to attract any buyer. Thus, $$p_{2}=0$$ is a best response to $$p_{1}=\varDelta +c$$ (charging more than 0 is also a best response, which, however, provides seller 1 with an incentive to increase its price). Therefore, $$\theta _{1}=1$$ yields a profit of $$r_{1}$$ if $$r_{2}\le c$$ and $$\varDelta +c$$ if $$r_{2}>c.$$

$\theta _{12}=1$ yields a profit of $\varDelta$ to the platform, which is less than that under $\theta _{1}=1$. Under $\theta _{2}=1$, the potential surplus is $r_{2}$ for front-page trading and $r_{1}-c$ for second-page trading. Thus, if $r_{2}\le c$, $\theta _{1}$ is optimal. Suppose $r_{2}>c$ and consider $\theta _{2}=1$.Then, the platform revenue cannot exceed $\left| \varDelta -c\right|$, because with the low search cost $c<r_{2}<r_{1}$, buyers can switch their seller, should that seller enjoy a profit exceeding $\left| \varDelta -c\right|$. Therefore, $\theta _{1}=1$ is optimal. $\square$

### *Proof*

*(Proposition* 3*)* Follow Fig. 1 for illustrative purposes. First observe that the profit function, $\pi (c)=\left( (1-G(c))^{2}+(G(c))^{2}\right) /g(c)$, is symmetric and U-shaped around $\bar{c}/2$. Since $c_{1}^{*}<c_{2}^{*}$, $c_{2}^{*}\le \bar{c}/2$ implies that $c_{1}^{*}<c_{2}^{*}<\bar{c}/2$. Thus, $\pi (c_{1}^{*})>\pi (c_{2}^{*})$ holds whenever $c_{2}^{*}\le \bar{c}/2$.

Suppose $c_{2}^{*}>\bar{c}/2$. By the symmetry of $g(\cdot )$, $G(c)=1-G(\bar{c}-c)$ and $g(c)=g(\bar{c}-c)$ for all *c*. Thus,

$$\begin{aligned} \frac{1-2G(c)}{g(c)}=\frac{1-G(c)-G(c)}{g(c)}=\frac{G(\bar{c}-c)-(1-G(\bar{c} -c))}{g(\bar{c}-c)}=-\frac{1-2G(\bar{c}-c)}{g(\bar{c}-c)}. \end{aligned}$$

This means that $Q(c)=(1-2G(c))/g(c)$ is an odd function around $\bar{c}/2$. See Fig. 1 to follow the logic. Let $Q^{+}(c)=Q(c)+\varDelta$ and $Q^{-}(c)=Q(c)-\varDelta$. By symmetry around $\bar{c}/2$, $Q(c)=-Q(\bar{c}-c)$. Therefore, $Q^{-}(c)=-Q^{+}(\bar{c}-c)$: one can rotate $Q^{+}(\cdot )$ about $(\bar{c}/2,0)$ by $180^{\circ }$ to get $Q^{-}(\cdot )$ in Fig. 1, and vice versa.

By definition, $c_{1}^{*}=Q^{-}(c_{1}^{*})$ and $c_{2}^{*}=Q^{+}(c_{2}^{*})$. Using symmetry to define $c_{2}^{*}$ from the “bottom” of the graph, $c_{2}^{*}- \bar{c}=Q^{-}(\bar{c}-c_{2}^{*})$. Thus, we can use $Q^{-}(c)$ to characterize both $c_{1}^{*}$ and $\bar{c}-c_{2}^{*}$.

$$c_{1}^{*} $$ lies at the intersection of $$Q^{-}$$ and a $$45^{\circ }$$ line; $$\bar{c} -c_{2}^{*}$$ lies at the intersection of $$Q^{-}$$ and line $$c-\bar{c}$$. Since line $$c-\bar{c}$$ is *below* the 45$$^{\circ }$$ line, it follows that $$c_{1}^{*}<\bar{c}-c_{2}^{*}$$. Moreover, $$c_{2}^{*}>\bar{c} /2$$ implies that the intersection lies left of the median: $$\bar{c} -c_{2}^{*}<\bar{c}/2$$. Thus, we have $$c_{1}^{*}<\bar{c}-c_{2}^{*}<\bar{c}/2$$, which, since $$\pi (c)$$ is decreasing for $$c<\bar{c}/2$$, implies

$$\begin{aligned} \pi (c_{1}^{*})>\pi (\bar{c}-c_{2}^{*})=\pi (c_{2}^{*}), \end{aligned}$$

where the equality holds because $\pi (c)$ is symmetric around $\bar{c}/2$. $\square$

## Footnotes

1 The model can be generalized to more than two sellers with a loss of clarity in proofs.

2 Table 1 comes from the Wikipedia entry *List of digital distribution platform for mobile devices*, retrieved on 7 Oct 2013, and assembled from the multiple sources cited there. Sellers on Amazon.com pay the higher of a per-item minimum fee or a fixed percentage of the sale price. The fixed percentage differs by category from 8 to 15% with some exceptions. eBay has similar pricing policies.

3 See reviews by Tirole (1988), Shapiro (1989), Cabral (2000), and Belleflamme and Peitz (2015).

4 See Rubinstein and Salant (2006) for the decision theory literature on the violation of the order irrelevance assumption.

5 The platform could charge the buyers a participation fee. Many platforms choose not to, precisely because there are many more buyers than sellers. Many platforms are free for buyers, and are not tied to any device or operating system (Amazon, iTunes, Steam), which rules out cross-subsidization or any other direct interest of the platform in the buyers’ satisfaction. A fixed fee to sellers would not change the predictions of our model, but in principle our model can be augmented for studying the effects on the endogenous entry of both buyers and sellers; and if this happens, the issue of participation fees will start to matter. In any case, Table 1 shows that these fees, even if they exist, are negligible for any serious enterprise.

6 Supermarkets also carry many products from many sellers and face similar product placement problems. However, our model is not immediately applicable to supermarkets because supermarkets, not sellers, set final good prices in this setting. Sellers still can compete with wholesale prices, but the structure of the problem changes significantly: there is no longer a disagreement between the price setter and the position setter.

7 However, it could be reasonable to assume that the operator charges similar fees to *actually* competing tenants, whose products are often similar. Even with different margins’ being paid to the operator by different sellers, without competition among operators, an operator’s choice would be simply biased towards the profit earned by whichever seller gives up the larger profit share, and the sellers’ behavior would not change. With competition, endogenizing margin choice would be an interesting issue, but that is beyond the scope of this study.

8 Marginal costs can be positive, and our analysis qualitatively does not change so long as the difference in marginal costs between the two products is not greater than the quality difference: $r_1-r_2$.

9 In this sense, “obfuscation” can be perceived as increasing the difficulty of making a purchase. See Ellison (2005) for examples of this.

10 With reverse timing, the arrangement of products would be determined by the prices that firms set. In situations where both firms earn positive profits, a small undercut to another firm will yield a change in positioning, which would result in a larger than first-order effect on the firm’s profits; this will lead to mixed strategy equilibria, although these do not seem to emerge in practice. Moreover, prices can be adjusted anytime in online stores; whereas product locations are not easy to change in physical stores. Thus, we focus on a situation in which a platform decides on product placement in anticipation that its placement will affect the degree of price competition.

11 We find multiple equilibria in the current framework, and therefore we use a robustness check of trembling hand perfection. Suppose that there is an equilibrium where no buyers visit the second page. Then the equilibrium is robust if the second-page seller’s behavior is optimal even when a small proportion of buyers visit the second page by mistake. This idea follows closely that of Stahl (1989).

12 The results would be the same qualitatively even if we design a more sophisticated model, for instance, based on Wolinsky (1986).

13 More precisely, to ensure uniqueness, it is sufficient to assume that $Q(c)=\frac{ 1-2G(c)}{g(c)}$ is decreasing, but we use the monotonicity of hazard rates to judge the ordering of prices.

14 The exact condition is $\varDelta <\left[ (1-G(c))^2+(G(c))^2\right] /g(c)$ for $c\in \{c_1^*,c_2^*\}$; this guarantees that showing both goods on the front page is dominated by showing only one product on the front page. Since $(1-x)^2+x^2>1/2 \ \forall x\in [0,1]$, $\varDelta <1/(2 g(c)) \ \forall c\in [0,\bar{c}]$ would be a sufficient condition.

15 We thank the referee for this example.

16 To replicate our analysis, the code available here can be used: https://sites.google.com/site/sergeyvpopov/papers/PlotProfits.m.

17 At the other end of the spectrum, we can allow for a continuum of preference types for buyers and show that the log-concavity of the distribution of types, along with symmetry, implies that the better product is put on the front page (Hsu et al. 2015).

18 As before, this analysis is based on the quitting-option irrelevance. As $\mu _{3}\rightarrow 0$, there is progressively less competition between products; hence showing both products on the front page can become optimal.
